# Estimation of Genetic Correlations of Primal Cut Yields with Carcass Traits in Hanwoo Beef Cattle

**DOI:** 10.3390/ani11113102

**Published:** 2021-10-30

**Authors:** Masoumeh Naserkheil, Deukmin Lee, Kihoon Chung, Mi Na Park, Hossein Mehrban

**Affiliations:** 1 Animal Breeding and Genetics Division, National Institute of Animal Science, Cheonan-si 31000, Korea; naserkheil@ut.ac.ir (M.N.); mina0412@korea.kr (M.N.P.); 2 Department of Animal Life and Environment Sciences, Hankyong National University, Jungang-ro 327, Anseong-si 17579, Korea; doctor9418@gmail.com; 3 Department of Animal Science, Shahrekord University, Shahrekord 88186-34141, Iran; hosseinmehrban@gmail.com

**Keywords:** genetic parameters, heritability, primal cut yields, carcass traits, Hanwoo

## Abstract

**Simple Summary:**

Recently, there is a growing interest in the genetic improvement of carcass merit in the Korean beef industry. Primal cut yields have been proposed to characterize the meat quality and quantity in beef cattle and are known to be genetically correlated with the carcass merit and premium prices. Hence, knowledge of genetic parameters is required to select the weight of primal cuts that may be used as the selection criteria for designing future breeding programs. This study aimed to estimate the heritability and genetic and phenotypic correlations of primal cut yields and carcass traits in Hanwoo cattle. All traits presented a medium to high heritability, which indicates a probable increase in their response to selection. In addition, moderate to highly favorable genetic correlations were found between the primal cut yields and carcass traits, such as the carcass weight and eye muscle area. Therefore, results suggested inclusion of primal cut traits as a selection objective in Hanwoo breeding programs to meet the growing demand for high quality products.

**Abstract:**

This study was carried out to estimate the variance components, heritability, and genetic correlations between the carcass traits and primal cut yields in Hanwoo cattle. Carcass traits comprising 5622 records included back fat thickness (BFT), carcass weight (CW), eye muscle area (EMA), and marbling score (MS). The 10 primal cut yields from 3467 Hanwoo steers included the tenderloin (TLN), sirloin (SLN), striploin (STLN), chuck (CHK), brisket (BSK), top round (TRD), bottom round (BRD), rib (RB), shank (SK), and flank (FK). In addition, three composite traits were formed by combining primal cut yields as novel traits according to consumer preferences and market price: high-value cuts (HVC), medium-value cuts (MVC), and low-value cuts (LVC). Heritability estimates for the interest of traits were moderate to high, ranging from 0.21 ± 0.04 for CHK to 0.59 ± 0.05 for MS. Except genetic correlations between RB and other primal cut traits, favorable and moderate to high correlations were observed among the yields of primal cut that ranged from 0.38 ± 0.14 (CHK and FK) to 0.93 ± 0.01 (TRD and BRD). Moreover, the estimated genetic correlations of CW and EMA with primal cut yields and three composite traits were positive and moderate to strong, except for BFT, which was negative. These results indicate that genetic progress can be achieved for all traits, and selection to increase the yields of primal cuts can lead to considerable profitability in the Hanwoo beef industry.

## 1. Introduction

In recent years, carcass and meat quality traits in beef cattle have received great attention, as they determine the profitability of the beef production systems. Meat quality is a comprehensive term used to describe the consumer acceptance of beef, as they are willing to pay more for higher-quality products [1]. In this respect, cattle breeders have paid more attention to increasing the lean meat content and marbling as well as quality grades that are consistent with consumer demands and preferences.

One of the famous breeds for carcass and meat quality traits is Hanwoo, an indigenous beef cattle in Korea [2]. Hanwoo beef is very popular in Korea because of its extensive marbling, tenderness, juiciness, and characteristic flavor [3], but its price is almost three times that of imported beef meat from other breeds [4]. In Hanwoo breeding program, important traits, such as the back fat thickness (BFT), carcass weight (CW), eye muscle area (EMA), and marbling score (MS), are commonly included in the selection criteria to increase the quality and quantity of meat [5]. As reviewed by Kim et al. [5], it was shown that an increasing trend of estimated breeding values for CW, EMA, and MS as well as a decreasing trend of estimated breeding values for BFT from 1985 to 2010 in Hanwoo cattle. Although substantial improvements have been obtained for carcass traits of Hanwoo cattle, due to market requirements for higher quality, other relevant traits have received less attention, such as primal cut yields, which influence both meat yield and quality, and continuous improvement of these invaluable traits is required. Primal cut yields are an important trait that command premium prices. It has been shown that high-value primal cuts have the potential to improve the economic value of animals at slaughter age [6,7]. In a previous study on Irish beef cattle, the possibility of genetically improving carcass value based on wholesale carcass cuts was reported [8].These traits have been proposed as indicators of carcass weight and have previously been reported to be moderately to strongly genetically correlated with carcass merit in beef cattle [6,9,10]. Moreover, the potential for improving the yield of individual primal cuts without increasing the overall carcass weight using breeding programs has been demonstrated [10]. Furthermore, the existence of genetic variability and moderate to high heritability for primal cuts has been demonstrated in Hanwoo cattle [6]. Hence, selection for the weight of primal cut yields requires knowledge of genetic parameters and their correlations with the carcass traits that are included in the Hanwoo selection index.

In addition to the assessment of primal cut yields in this study, three composite traits were obtained from the primal cut yields according to the consumer preference and price. To this end, we estimated the genetic parameters of primal cut yields and three composite traits as well as the genetic and phenotypic correlations between these traits and carcass traits in Hanwoo cattle.

## 2. Materials and Methods

### 2.1. Animals and Phenotypes

Data used in this study were collected from the Hanwoo Improvement Center (HIC) of the National Agricultural Cooperative Federation, Korea. Carcass traits were available on 5622 Hanwoo steers (born between 2003 and 2017), of which 3467 steers had records for primal cut traits (born between 2008 and 2017). All animals participate in the progeny test program for selecting proven bulls under the Korean National Hanwoo Cattle Improvement System. The steers were castrated around 4 to 5 months of age. The batch, which represented the contemporary group of the animal, was two batches in each production year. Further details have been previously described by Kim et al. [5]. The pedigree data comprised 60,060 animals after tracing the pedigree file back 11 generations. Four carcass traits analyzed were BFT, CW, EMA, and MS, which were collected from steers at approximately 24 months of age according to the Korean carcass grading system. CW was recorded for each animal after chilling for approximately 24 h postmortem, whereas BFT, EMA, and MS were measured on the longissimus dorsi muscle between the 13th rib and the first lumbar vertebra according to notification No. 2014-4 of the Ministry of Agriculture, Food and Rural Affairs. MS was graded based on a categorical system of nine classes that range from 1 (no marbling) to 9 (abundant marbling). Primal cut yields are composed of both unique and composite meat cuts from the forequarter and hindquarter. The 10 traits analyzed were tenderloin (TLN), sirloin (SLN), striploin (STLN), chuck (CHK), brisket (BSK), top round (TRD), bottom round (BRD), rib (RB), shank (SK), and flank (FK). The locations of each cut on the carcass are shown in Figure S1. Three composite traits were defined as traits that are not commonly used for genetic parameter estimation or included in selection indices and were made according to the consumer preference and market price as follows: high-value cuts (HVC: TLN, SLN, STLN, and CHK), medium-value cuts (MVC: BSK, TRD, BRD, and RB), and low-value cuts (LVC: SK and FK). The plot and distribution for the traits of interest were surveyed for the exclusion of unreasonable data as outliers, which resulted in elimination of 2 records on average for each trait. The slaughter dates of steers were between 11 May 2005 and 14 November 2019 for carcass traits (274 levels) and 5 November 2010 to 16 May 2019 for primal cut yields (180 levels). Table 1 shows the descriptive statistics for each trait.

### 2.2. Statistical Analysis

The genetic and phenotypic correlations among the traits of interest were estimated using a series of bivariate pedigree-based animal models with the average-information algorithm implemented in the AIREMLF90 software [11] as follows:

(1) y=Xb+Zu+e 

where **y** is the vector of phenotypic observations; **b** is the vector representing the fixed effects, comprising the slaughter date (180 levels) and slaughter age (days from birth to slaughter) was considered as covariates for carcass traits; **u** is the vector of random genetic additive effects, and **e** is the vector of random residual effects. The **X** and **Z** are incidence matrices that relate the records to the fixed and random genetic additive effects, respectively.

The random effects of **u** and **e** were assumed to follow the multivariate normal distribution with the mean of zero and Var (**u**) = **G**⊗**A** and Var(**e**) = **R**⊗**I**, in which **A** is the numerator relationship matrix, **I** is the identity matrix, and **G** and **R** are additive genetic and residual covariance, respectively; and ⊗ is the Kronecker product.

The variance components and heritabilities were estimated using a single-trait pedigree-based animal model with the same fixed effects as in Equation (1).

The convergence criterion for estimation of variance components was computed according to the formula [12]:

$$C=\frac{\sum_{i}{\left(\theta_{i}-{\;\theta }_{i}^{*}\right)}^{2}}{\sum_{i}\theta_{i}^{2}}$$

where $\theta_{i}$ and $\theta_{i}^{*}$ are the current and previous estimate of ith variance component, respectively. The iteration stops if C is less than 10^−12^ for all evaluations as a default of program.

In addition, the genetic (r_g_) and phenotypic (r_p_) correlations were obtained as:

$$r_{g}=\frac{\sigma_{g_{X,Y}}}{\sqrt{\sigma_{g_{X}}^{2}\sigma_{g_{Y}}^{2}}},{\;r}_{p}=\frac{\sigma_{p_{X,Y}}}{\sqrt{\sigma_{p_{X}}^{2}\sigma_{p_{Y}}^{2}}}$$

where, $\sigma_{g_{X,Y}}$ and $\sigma_{p_{X,Y}}$ are genetic and phenotypic covariance between traits X and Y, respectively;${\;\sigma }_{g}^{2}$ is additive genetic variance and $\sigma_{p}^{2}\;$ is phenotypic variance of the corresponding traits.

Furthermore, coefficient of genetic variation (CV_g_) was defined as the square root of the additive genetic variance divided by the mean of the trait (${\mathrm{CV}}_{g}\%=\frac{\sigma_{g}}{\bar{x}}$ × 100).

The standard error for the (co)variance components, correlations and heritabilities was estimated by 10,000 times repetitive sampling with the option “se_covar_function” in AIREMLF90 software [11].

In order to determine significant of genetic and phenotypic correlations different from zero, the full and reduced models were compared by the chi-squared distribution of -2log-likelihood estimates as follows [13]:

$$\Lambda =\frac{{\mathrm{likelihood}}_{\mathrm{reduced}\;\mathrm{model}}}{{\mathrm{likelihood}}_{\mathrm{full}\;\mathrm{model}}},-2\log\Lambda =-2\log\left({\mathrm{likelihood}}_{\mathrm{reduced}\;\mathrm{model}}-{\mathrm{likelihood}}_{\mathrm{full}\;\mathrm{model}}\right)$$

The genetic and residual covariances with non-zero values were considered in the full model; however, the matrix **G** was diagonal in reduced model. The genetic correlation was significant when −2logΛ was more than 3.84 (the chi-squared statistic with one degree of freedom and α equal 0.05). Similarly, the significance of phenotypic correlation was tested; however, the **G** and **R** matrices (in the reduced model) were diagonal and the chi-squared statistic with two degree of freedom and α equal 0.05 was 5.99 (shown in Supplementary Material File S1).

## 3. Results and Discussion

### 3.1. Heritability Estimates

Knowledge of genetic parameters, along with phenotypic and pedigree information, is a prerequisite to carry out genetic evaluations, design breeding schemes, and obtain indices to increase the response to selection. Hence, this study was performed to estimate the heritability and variance components of primal cut yields and their correlations with carcass traits in Hanwoo cattle. The estimates of the heritability and variance components with standard errors for all traits are given in Table 2. Estimates of heritability were from moderate to high for the studied traits, which ranged from 0.21 ± 0.04 to 0.59 ± 0.05 (Table 2), representing that gains by direct selection are feasible for the majority of the traits. Heritability estimates for the carcass traits were 0.57 ± 0.05 (BFT), 0.28 ± 0.04 (CW), 0.46 ± 0.05 (EMA), and 0.59 ± 0.05 (MS), being comparable to those obtained in Hanwoo [6,14,15,16,17,18,19,20], Japanese Black [21,22], Simmental [23], Brahman [24], and Nellore cattle [25,26]. The 10 primal cut traits had moderate to high heritabilities, being the highest (0.52 ± 0.06) and the lowest (0.21 ± 0.04) for TRD and CHK, respectively. Heritability estimates for other primal cut yields, including TLN, SLN, STLN, BSK, BRD, SK, FK, and RB, were 0.34 ± 0.05, 0.42 ± 0.06, 0.39 ± 0.06, 0.51 ± 0.06, 0.50 ± 0.06, 0.50 ± 0.06, 0.29 ± 0.05, and 0.27 ± 0.05, respectively. According to the literature, only a few studies have estimated the genetic parameters for individual primal cut yields. In addition, comparing these estimates with values reported in the literature is difficult because the definition of primal cuts differs between various researches. In a previous study on Hanwoo cattle, Choi et al. [6] reported higher heritability for TLN (0.41), SLN (0.60), STLN (0.64), TRD (0.62), BRD (0.66), and RB (0.35); lower heritability for BSK (0.21), SK (0.35), and FK (0.21); and slightly similar heritability for CHK (0.19). The differences between our estimates and those obtained by Choi et al. [6] could be due to the discrepancy in the number of records measured (3467 vs. 920 records) and the unit of measurement data (kilogram vs. percent of carcass weight). Moreover, another study reported higher heritability for TLN (0.49), SLN (0.50), STLN (0.51), CHK (0.34), TRD (0.70), BRD (0.71), FK (0.38), and RB (0.41), and lower heritability for BSK (0.38) and SK (0.32) than the estimates obtained in the present study for Hanwoo cattle [27]. In comparison with other breeds, our estimates of heritability for most primal cut yields were comparable to previously reported values in the Irish cattle [28]. They estimated a heritability of 0.55, 0.41, 0.41, 0.47, 0.42, 0.37, and 0.28 for SLN, STLN, CHK, BSK, BRD, FK, and RB, respectively. Similarly, Judge et al. [10] and Berry et al. [9] reported heritability estimates of 0.30, 0.51, 0.39, and 0.68 for STLN, CHK, BSK, and SK in Irish cattle, respectively, which are inconsistent with the present findings. The estimated heritability of STLN was similar to obtained value of 0.40 by Moor et al. [29] for the UK beef cattle, but higher than the estimate of 0.24 reported by Zhu et al. [30] for the Chinese Simmental cattle. Additionally, Zhu et al. [30] estimated heritability of 0.39 and 0.27 for TLN and CHK, respectively, which were higher than our estimates. Another study also showed moderate to high heritability estimates for some primal cut traits, ranging from 0.21 to 0.74 in Chianina cattle [31]. Furthermore, in other earlier studies, Cundiff et al. [32] reported heritability estimates for ribs (0.38 to 0.44), loins (0.07 to 0.48), and round cut (0.42 to 0.68), while Brackelsberg et al. [33] estimated a high heritability (0.81) for the composite cut called round and loin, which were comparable to our findings.

Heritability estimates for the composite traits, HVC, MVC, and LVC, were 0.34 ± 0.05, 0.35 ± 0.05, and 0.36 ± 0.06, respectively, and were comparable to those reported by Pabiou et al. [8] in Irish steers (0.13 to 0.37). To the best of our knowledge, this is the first study on composite primal cut yields in Hanwoo cattle according to consumer preferences and price. In a recent study, primal cut yields were combined into group cuts that included frying cuts, roasting cuts, and mince cuts with heritability estimates of 0.42, 0.73, and 0.46, respectively [10].

The favorable and moderate to high heritability of primal cut yields demonstrate that direct selection may have a considerable influence on these traits. Overall, the discrepancy in the estimates of present study with those of previous studies may be due to differences in slaughter age of animals considered, the number of records, breed, completeness of the pedigree, and statistical models used for analyses. The results also showed considerable additive genetic variation (based on CV_g_) for BFT (27.05%) and MS (33.99%), while the other traits had relatively low variations (4.68 to 9.23%). The magnitude of genetic variability in a trait determines its evolvability [34] and, consequently, can affect the capacity to change traits through breeding. In other words, the expected genetic gain (with the standardized scale) will be higher for BFT and MS relative to other traits.

In addition, our results showed that the genetic parameters obtained using bivariate models were close to the corresponding univariate models (Table S1 and Supplementary Material File S2).

### 3.2. Genetic and Phenotypic Correlations

Table 3 indicates the results of the genetic and phenotypic correlations among the 10 primal cut traits. The estimates for the genetic correlation between primal cut yields were low to high and positive, which ranged from 0.16 ± 0.15 to 0.93 ± 0.01. Our results showed that the highest genetic correlation was obtained between TRD and BRD (0.93 ± 0.01), followed by the correlation between BSK and BRD (0.92 ± 0.02), and between BRD and SK (0.91 ± 0.02). The estimated genetic correlation between the yields of most primal cuts was moderate to high and positive, except for RB, which had low to moderate correlations with other traits. Moreover, the phenotypic correlation estimates among the primal cut yields varied from very low (0.09 ± 0.02) to strong (0.90 ± 0.00), which showed the same trend as the genetic correlations. Similar findings have been achieved in Hanwoo cattle by Choi et al. [6] and Al-Mamun et al. [27] who reported positive genetic correlations between most primal cut yields, with the exception of the correlations of RB, which were negative and low to high. Consistent with our results, Brackelsberg et al. [33] indicated that genetic correlations between the four studied cuts (chuck and rib, round and loin; and round, loin, and rib) were relatively low to strong, which ranged from 0.16 to 1.00. Similarly, Cundiff et al. [32] found strong genetic correlations (0.72+) between primal cut yields, namely round, loin, rib, and chuck. Pabiou et al. [28] also showed relatively moderate to strong positive genetic correlations for all eight primal cuts, including striploin, sirloin, round, chuck, blade, brisket, rib, and fillet from commercial data set in Irish beef cattle, ranging from 0.35 to 0.85. In the study performed by Sarti et al. [31], high and positive genetic correlations (0.64 to 0.95) were observed between certain cuts (brisket, fore shank, chuck, rib, short plate, round, and loin) in Chianina cattle, which were higher than the values reported in the present study for the same traits. In addition, strong and positive, almost unity genetic correlations (0.92+) were estimated by Moor et al. [29] between the primal cut yields, namely, topside, silverside, striploin, fillet, knuckle, and rump in the UK beef cattle. Recently, Judge et al. [10] working on Irish cattle reported moderate to strong and positive genetic correlations (mean correlation of 0.72, with all correlations being ≥0.37) among 14 primal cut weights, which were in agreement with those estimated in our study for similar traits.

Results of the genetic correlations among the primal cuts and carcass traits showed positive, and relatively moderate to high correlations for all the primal cut yields with both CW and EMA and varied from 0.34 ± 0.10 to 0.85 ± 0.04, respectively (Table 4). The genetic correlations of MS with the primal cut yields were very low or near zero to relatively moderate, ranging from 0.02 ± 0.10 to 0.44 ± 0.08, indicating that given the absence of a genetic correlation between MS and these traits, selection for greater marbling is feasible. However, genetic correlation between BFT and the primal cuts was negative and relatively moderate, except for the correlation with RB, which had a very low, positive magnitude of 0.10 ± 0.11. The phenotypic correlations of carcass traits with the primal cut yields also indicated the same tendency as the genetic correlations but were higher for CW and lower magnitude for other carcass traits (i.e., EMA, BFT, and MS), ranging from almost zero to strong. Similar or higher estimates of the genetic correlation between CW and different primal cut yields were reported by Pabiou et al. [28] (0.45 to 0.67) and Judge et al. [10] (0.77 to 0.93) for Irish cattle, by Sarti et al. [31] for Chianina cattle (0.68 to 0.88), and by Moor et al. [29] for UK beef cattle (0.91 to 0.95). Moreover, the genetic correlation of BFT with primal cut yields in the current study was comparable to those estimated magnitudes of −0.10 to −0.59 for Irish cattle [28], −0.16 to −0.29 for UK beef cattle [29], and −0.18 to −0.71 for Hanwoo cattle [6]. This indicates that selection for decreasing the BFT improves the primal cut yields. Concerning the correlation between EMA and primal cut traits, there was a reasonable agreement between estimates from our study with those obtained in a previous study in Hanwoo cattle [6]. Overall, these results indicate that selection for greater carcass weight will lead to an increase in the weights of various primal cuts, whereas it is expected that selection to increase the weights considered in conjunction with other carcass traits, such as EMA.

Table 5 presents the genetic and phenotypic correlations among the composite primal cut and carcass traits. In comparison to the other carcass traits, genetic correlations between CW and the three composite traits (HVC, MVC, and LVC) were positive and strong, and ranged from 0.71 ± 0.06 to 0.92 ± 0.02, followed by the correlation between HVC and EMA (0.81 ± 0.05). BFT showed negative, relatively moderate genetic correlations with HVC, MVC, and LVC (−0.40 ± 0.09, −0.24 ± 0.10, −0.30 ± 0.10, respectively), while the genetic correlations of MS with HVC (0.33 ± 0.09), MVC (0.24 ± 0.10), and LVC (−0.03 ± 0.10) were nearly zero to low. In addition, the phenotypic correlations of CW with all the composite traits were positive and strong (0.69 ± 0.01 to 0.94 ± 0.00). Phenotypically, EMA was moderately positive correlated with HVC (0.63 ± 0.01), MVC (0.55 ± 0.01), and LVC (0.42 ± 0.02). Conversely, BFT and MS showed near zero to very low phenotypic correlations with all the composite traits, which ranged from −0.02 ± 0.02 to 0.10 ± 0.02 for BFT and from −0.13 ± 0.02 to 0.14 ± 0.02 for MS. Similar results have been indicated by Pabiou et al. [8] who estimated moderate genetic correlations between the four wholesale cut weights (i.e., very high value cuts, high value cuts, medium value cuts, and low value cuts) and carcass weight in Irish steers, ranging from 0.32 between medium value cuts and CW to 0.45 between CW and very high value cuts. Hence, based on existing moderate to high heritability as well as favorable genetic correlation among carcass traits, composite traits, and primal cut yields, it can be mentioned that the potential benefits of heavier carcass or carcass with a greater proportion of higher value cuts can be easily elucidated, thereby aiding in decision-making about selection for carcass traits and high-value cuts to obtain a desired genetic response.

## 4. Conclusions

Our results indicate that the carcass and primal cut yields are heritable, and the existence of sufficient genetic variation can lead to improvements in these traits through genetic selection. Genetic correlations of primal cut yields with CW and EMA were moderate to highly favorable, while low to moderate and negative correlations between the primal cut traits with MS and BFT were observed. In addition, among the composite primal cut and carcass traits, such as CW and EMA, there were relatively strong and favorable correlations, representing that the genetic evaluation of carcass traits could be improved by utilization of primal cut yields as potential indicators for carcass merit. Consequently, the inclusion of these traits by breeders as selection criteria can provide a good opportunity for developing a selection index combining the primal cut yields and carcass traits in order to increase the response to selection and identification of candidate animals and it can result in substantial profitability of production systems.

## Figures and Tables

**Table 1:** Summary statistics for carcass traits, primal cut yields, and composite traits in Hanwoo cattle.

Trait (Unit)	Number of Records	Mean (SE)	Min.	Max.	SD	CV (%)
slaughter age (month)	5626	23.71 (0.01)	21.40	25.51	0.64	2.71
**Carcass traits**						
CW (kg)	5619	370.48 (0.57)	213.00	562.00	42.80	11.55
EMA (cm^2^)	5617	81.62 (0.12)	50.00	121.00	8.98	11.00
BFT (mm)	5622	9.92 (0.05)	1.00	35.00	3.95	39.83
MS (score)	5622	3.53 (0.02)	1.00	9.00	1.64	46.50
**Primal cut yields**						
TLN (kg)	3466	6.04 (0.01)	3.00	9.00	0.76	12.65
SLN (kg)	3465	34.23 (0.07)	16.80	50.70	4.11	12.02
STLN (kg)	3465	7.85 (0.02)	4.30	12.40	1.17	14.96
CHK (kg)	3463	14.61 (0.06)	6.70	34.80	3.76	25.72
BSK (kg)	3466	23.76 (0.05)	12.60	38.60	3.01	12.67
TRD (kg)	3467	20.22 (0.04)	10.50	30.20	2.43	12.00
BRD (kg)	3467	32.99 (0.07)	16.60	49.60	3.92	11.89
SK (kg)	3466	14.66 (0.03)	9.00	21.70	1.77	12.09
FK (kg)	3465	28.29 (0.08)	12.50	50.30	4.83	17.08
RB (kg)	3467	57.55 (0.13)	21.70	89.30	7.53	13.09
**Composite traits**						
HVC (kg) ^1^	3459	62.71 (0.13)	33.60	98.80	7.94	12.66
MVC (kg) ^2^	3464	134.53 (0.26)	71.60	188.00	15.2	11.30
LVC (kg) ^3^	3463	42.96 (0.10)	23.00	70.70	6.09	14.18

CW, carcass weight; BFT, backfat thickness; EMA, eye muscle area; MS, marbling score; TLN, tenderloin; SLN, sirloin; STLN, striploin; CHK, chuck; BSK, brisket; TRD, top round; BRD, bottom round; SK, shank; FK, flank; RB, rib; ^1^ HVC, (CHK+SLN+STLN+TLN); ^2^ MVC, (BSK+TRD+BRD+RB); ^3^ LVC, (FK+SK); SD, standard deviation; SE, standard error that is SD divided by the root of n (number of records); CV, coefficient of variation that is the standard deviation divided by mean.

**Table 2:** Estimates of heritability (h^2^), additive genetic variance (σ^2^_g_), residual variance (σ^2^_e_), and coefficient of genetic variation (CV_g_) for carcass traits, primal cut yields, and composite traits in Hanwoo cattle.

Trait	h^2^	σ^2^_g_	σ^2^_e_	σ^2^_p_	CV_g_ (%)
**Carcass traits**					
CW	0.28 (0.04)	303.64 (45.29)	783.07 (40.03)	1086.70 (22.51)	4.68
EMA	0.46 (0.05)	29.06 (3.27)	33.58 (2.63)	62.64 (1.39)	6.60
BFT	0.57 (0.05)	7.20 (0.72)	5.48 (0.56)	12.68 (0.29)	27.05
MS	0.59 (0.05)	1.44 (0.14)	1.01 (0.11)	2.45 (0.06)	33.99
**Primal cut yields**					
TLN	0.34 (0.05)	0.14 (0.02)	0.27 (0.02)	0.42 (0.01)	6.19
SLN	0.42 (0.06)	5.26 (0.78)	7.20 (0.65)	12.46 (0.34)	6.70
STLN	0.39 (0.06)	0.31 (0.05)	0.50 (0.04)	0.81 (0.02)	7.09
CHK	0.21 (0.04)	1.82 (0.38)	6.64 (0.36)	8.46 (0.22)	9.23
BSK	0.51 (0.06)	3.17 (0.42)	3.08 (0.34)	6.25 (0.18)	7.49
TRD	0.52 (0.06)	2.22 (0.29)	2.07 (0.23)	4.29 (0.12)	7.37
BRD	0.50 (0.06)	5.47 (0.73)	5.41 (0.59)	10.87 (0.30)	7.09
SK	0.50 (0.06)	1.10 (0.15)	1.11 (0.12)	2.20 (0.06)	7.15
FK	0.29 (0.05)	4.61 (0.86)	11.58 (0.77)	16.18 (0.42)	7.59
RB	0.27 (0.05)	9.58 (1.93)	27.18 (1.75)	37.04 (0.96)	5.38
**Composite traits**					
HVC ^1^	0.34 (0.05)	15.63 (2.57)	30.02 (2.23)	45.65 (1.21)	6.30
MVC ^2^	0.35 (0.05)	51.44 (8.39)	93.57 (7.22)	145.01 (3.87)	5.33
LVC ^3^	0.36 (0.06)	9.14 (1.50)	16.59 (1.29)	25.72 (0.69)	7.04

CW, carcass weight; BFT, backfat thickness; EMA, eye muscle area; MS, marbling score; TLN, tenderloin; SLN, sirloin; STLN, striploin; CHK, chuck; BSK, brisket; TRD, top round; BRD, bottom round; SK, shank; FK, flank; RB, rib; ^1^ HVC, (CHK+SLN+STLN+TLN); ^2^ MVC, (BSK+TRD+BRD+RB); ^3^ LVC, (FK+SK). The numbers in parentheses are standard errors.

**Table 3:** Estimates of genetic (above diagonal) and phenotypic (below diagonal) correlations (standard error (SE) in parentheses) among the primal cut yields in Hanwoo cattle.

Trait	TLN	SLN	STLN	CHK	BSK	TRD	BRD	SK	FK	RB
TLN	1.00	0.62 (0.07)	0.57 (0.08)	0.54 (0.10)	0.85 (0.04)	0.79 (0.04)	0.86 (0.04)	0.82 (0.04)	0.58 (0.09)	**0.18** (0.13)
SLN	0.68 (0.01)	1.00	0.85 (0.04)	0.60 (0.09)	0.76 (0.04)	0.64 (0.06)	0.72 (0.05)	0.72 (0.05)	0.68 (0.07)	0.52 (0.09)
STLN	0.66 (0.01)	0.73 (0.01)	1.00	0.52 (0.11)	0.73 (0.05)	0.67 (0.06)	0.74 (0.05)	0.75 (0.05)	0.63 (0.08)	0.43 (0.10)
CHK	0.49 (0.01)	0.55 (0.01)	0.44 (0.01)	1.00	0.69 (0.07)	0.64 (0.08)	0.63 (0.08)	0.58 (0.09)	0.38 (0.14)	**0.16** (0.15)
BSK	0.74 (0.01)	0.78 (0.01)	0.66 (0.01)	0.56 (0.01)	1.00	0.88 (0.03)	0.92 (0.02)	0.87 (0.03)	0.68 (0.07)	0.39 (0.10)
TRD	0.74 (0.01)	0.72 (0.01)	0.66 (0.01)	0.53 (0.01)	0.81 (0.01)	1.00	0.93 (0.01)	0.83 (0.03)	0.72 (0.06)	**0.21** (0.11)
BRD	0.77 (0.01)	0.76 (0.01)	0.70 (0.01)	0.53 (0.01)	0.85 (0.01)	0.90 (0.00)	1.00	0.91 (0.02)	0.74 (0.06)	0.33 (0.10)
SK	0.71 (0.01)	0.71 (0.01)	0.65 (0.01)	0.48 (0.01)	0.82 (0.01)	0.82 (0.01)	0.86 (0.00)	1.00	0.76 (0.06)	0.36 (0.10)
FK	0.49 (0.01)	0.56 (0.01)	0.51 (0.01)	0.09 (0.02)	0.57 (0.01)	0.61 (0.01)	0.62 (0.01)	0.62 (0.01)	1.00	0.45 (0.11)
RB	0.50 (0.01)	0.68 (0.01)	0.54 (0.01)	0.34 (0.02)	0.57 (0.01)	0.49 (0.01)	0.56 (0.01)	0.53 (0.01)	0.47 (0.01)	1.00

TLN, tenderloin; SLN, sirloin; STLN, striploin; CHK, chuck; BSK, brisket; TRD, top round; BRD, bottom round; SK, shank; FK, flank; RB, rib. The bold numbers represent non-significant values (*p* > 0.05).

**Table 4:** Estimates of genetic and phenotypic correlations (SE in parentheses) among the primal cut yields and carcass traits in Hanwoo cattle.

Trait	TLN	SLN	STLN	CHK	BSK	TRD	BRD	SK	FK	RB
**G** **enetic** **correlations**										
CW	0.55 (0.08)	0.73 (0.05)	0.67 (0.06)	0.47 (0.11)	0.73 (0.05)	0.60 (0.06)	0.74 (0.05)	0.72 (0.05)	0.64 (0.08)	0.82 (0.04)
EMA	0.45 (0.08)	0.77 (0.05)	0.85 (0.04)	0.58 (0.10)	0.59 (0.06)	0.54 (0.07)	0.55 (0.07)	0.52 (0.07)	0.39 (0.10)	0.34 (0.10)
BFT	−0.46 (0.09)	−0.36 (0.09)	−0.30 (0.09)	−0.33 (0.11)	−0.36 (0.08)	−0.43 (0.08)	−0.33 (0.08)	−0.32 (0.08)	−0.26 (0.10)	**0.10** (0.11)
MS	**0.02** (0.10)	0.44 (0.08)	0.31 (0.09)	**0.06** (0.12)	**0.16** (0.09)	**0.03** (0.09)	**0.05** (0.09)	**0.03** (0.09)	**−0.06** (0.10)	0.43 (0.10)
**Phenotypic correlations**										
CW	0.67 (0.01)	0.83 (0.01)	0.70 (0.01)	0.51 (0.01)	0.78 (0.01)	0.72 (0.01)	0.79 (0.01)	0.75 (0.01)	0.59 (0.01)	0.86 (0.00)
EMA	0.48 (0.01)	0.63 (0.01)	0.66 (0.01)	0.39 (0.02)	0.51 (0.01)	0.53 (0.01)	0.54 (0.01)	0.48 (0.01)	0.36 (0.02)	0.40 (0.02)
BFT	−0.06 (0.02)	−0.01 (0.02)	0.01 (0.02)	−0.04 (0.02)	−0.07 (0.02)	−0.10 (0.02)	−0.06 (0.02)	−0.07 (0.02)	**0.01** (0.02)	0.30 (0.02)
MS	**0.02** (0.02)	0.21 (0.02)	0.17 (0.02)	**0.00** (0.02)	**0.01** (0.02)	−0.08 (0.02)	**−0.02** (0.02)	−0.07 (0.02)	−0.14 (0.02)	0.22 (0.02)

CW, carcass weight; BFT, backfat thickness; EMA, eye muscle area; MS, marbling score; TLN, tenderloin; SLN, sirloin; STLN, striploin; CHK, chuck; BSK, brisket; TRD, top round; BRD, bottom round; SK, shank; FK, flank; RB, rib. The bold numbers represent non-significant values (*p* > 0.05).

**Table 5:** Estimates of genetic and phenotypic correlations (SE in parentheses) between the composite primal cut traits and carcass traits in Hanwoo cattle.

Trait	CW	EMA	BFT	MS
**Genetic correlations**				
HVC ^1^	0.73 (0.05)	0.81 (0.05)	−0.40 (0.09)	0.33 (0.09)
MVC ^2^	0.92 (0.02)	0.59 (0.07)	−0.24 (0.10)	0.24 (0.10)
LVC ^3^	0.71 (0.06)	0.45 (0.09)	−0.30 (0.10)	**−0.03** (0.10)
**Phenotypic correlations**				
HVC	0.81 (0.01)	0.63 (0.01)	−0.02 (0.02)	0.14 (0.02)
MVC	0.94 (0.00)	0.55 (0.01)	0.10 (0.02)	0.09 (0.02)
LVC	0.69 (0.01)	0.42 (0.02)	−0.02 (0.02)	−0.13 (0.02)

CW, carcass weight; BFT, backfat thickness; EMA, eye muscle area; MS, marbling score; ^1^ HVC, (CHK+SLN+STLN+TLN); ^2^ MVC, (BSK+TRD+BRD+RB); ^3^ LVC, (FK+SK). The bold numbers represent non-significant values (*p* > 0.05).

## Data Availability

The data presented in this study are available at http://www.limc.co.kr and http://www.ekape.or.kr (accessed on 11 February 2021).

## Supplementary Materials

The following are available online at https://www.mdpi.com/article/10.3390/ani11113102/s1, Figure S1: Location of 10 carcass primal cut yields in Hanwoo cattle; Table S1: The average of estimates of heritability (h^2^), additive genetic variance (σ^2^_g_), residual variance (σ^2^_e_), and phenotypic variance (σ^2^_p_) for carcass traits, primal cut yields, and composite traits using bivariate models in Hanwoo cattle; Supplementary Material File S1: Estimates of -2loglikelihoods for investigating significantly different from zero of genetic and phenotypic correlations; Supplementary Material File S2: The genetic parameters obtained using bivariate models.
