# Supplementary material for: Estimation of Genetic Correlations of Primal Cut Yields with Carcass Traits in Hanwoo Beef Cattle
Source: Animals (Basel). 2021 Oct 30;11(11):3102. doi: 10.3390/ani11113102 (PMC8614487; doi:10.3390/ani11113102)
Supplement: Supplementary file 1 [file animals-11-03102-s001.zip › Supplementary Table S1.pdf]

Table S1. The average of estimates of heritability (h^2), additive genetic variance (σ^2_g), residual variance (σ^2_e), and phenotypic variance (σ^2_p) for carcass traits, primal cut yields, and composite traits using bivariate models in Hanwoo cattle.

Trait	h^2	σ^2_g	σ^2_e	σ^2_p
		**Carcass traits**		
CW	0.28	304.63	783.36	1087.99
EMA	0.47	29.23	33.48	62.70
BFT	0.58	7.31	5.40	12.71
MS	0.59	1.44	1.01	2.45
		**Primal cut yields**		
TLN	0.35	0.14	0.27	0.42
SLN	0.43	5.32	7.15	12.47
STLN	0.39	0.31	0.50	0.81
CHK	0.21	1.80	6.66	8.46
BSK	0.51	3.18	3.06	6.24
TRD	0.52	2.23	2.05	4.28
BRD	0.50	5.48	5.38	10.86
SK	0.50	1.10	1.10	2.20
FK	0.29	4.63	11.54	16.18
RB	0.27	9.88	27.10	36.99
		**Composite traits**		
HVC [1]	0.34	15.76	30.04	45.80
MVC [2]	0.36	51.6	93.23	144.83
LVC [3]	0.36	9.16	16.54	25.70

CW, carcass weight; BFT, backfat thickness; EMA, eye muscle area; MS, marbling score; TLN, tenderloin; SLN, sirloin; STLN, striploin; CHK, chuck; BSK, brisket; TRD, top round; BRD, bottom round; SK, shank; FK, flank; RB, rib; [1] HVC, (CHK+SLN+STLN+TLN); [2] MVC, (BSK+TRD+BRD+RB); [3] LVC, (FK+SK).
