# Supplementary figures and images for: Estimation of Genetic Correlations of Primal Cut Yields with Carcass Traits in Hanwoo Beef Cattle
Source: Animals (Basel). 2021 Oct 30;11(11):3102. doi: 10.3390/ani11113102 (PMC8614487; doi:10.3390/ani11113102)

Supplementary Figure

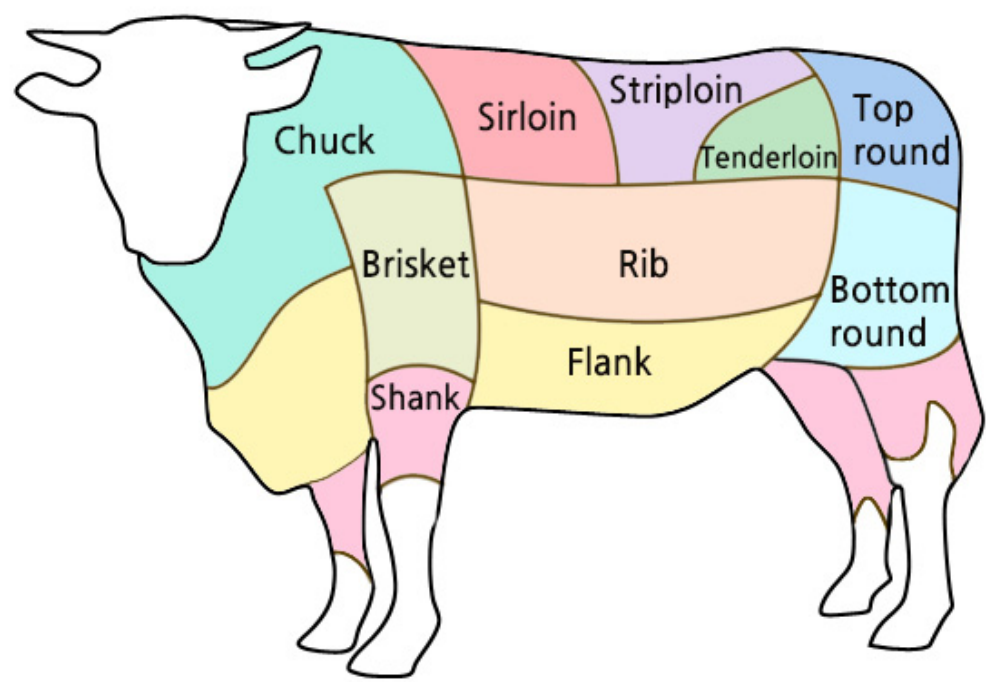

**Figure S1.** Location of 10 carcass primal cut yields in Hanwoo cattle.

Supplement: Supplementary file 1 [file animals-11-03102-s001.zip › Supplementary Figure S1.pdf]
